# The impact of COVID-19 on eye health disparities: uncovering and addressing critical challenges

**Published:** 2024-10-02

**Authors:** Anthea Burnett, Amanda Davis

**Affiliations:** 1 Conjoint Lecturer: School of Optometry and Vision Science, The University of New South Wales, Sydney, Australia.; 2 Knowledge Consultant: International Agency for the Prevention of Blindness.; 3 Regional Chair, Western Pacific: International Agency for the Prevention of Blindness and Director of Strategic Initiatives: The Fred Hollows Foundation, Sydney, Australia.

**The COVID-19 pandemic caused a substantial disruption in global health care services, leading to significant eye health backlogs that may take years to clear, depending on the region and the capacity of the health care system.**

**Figure:**
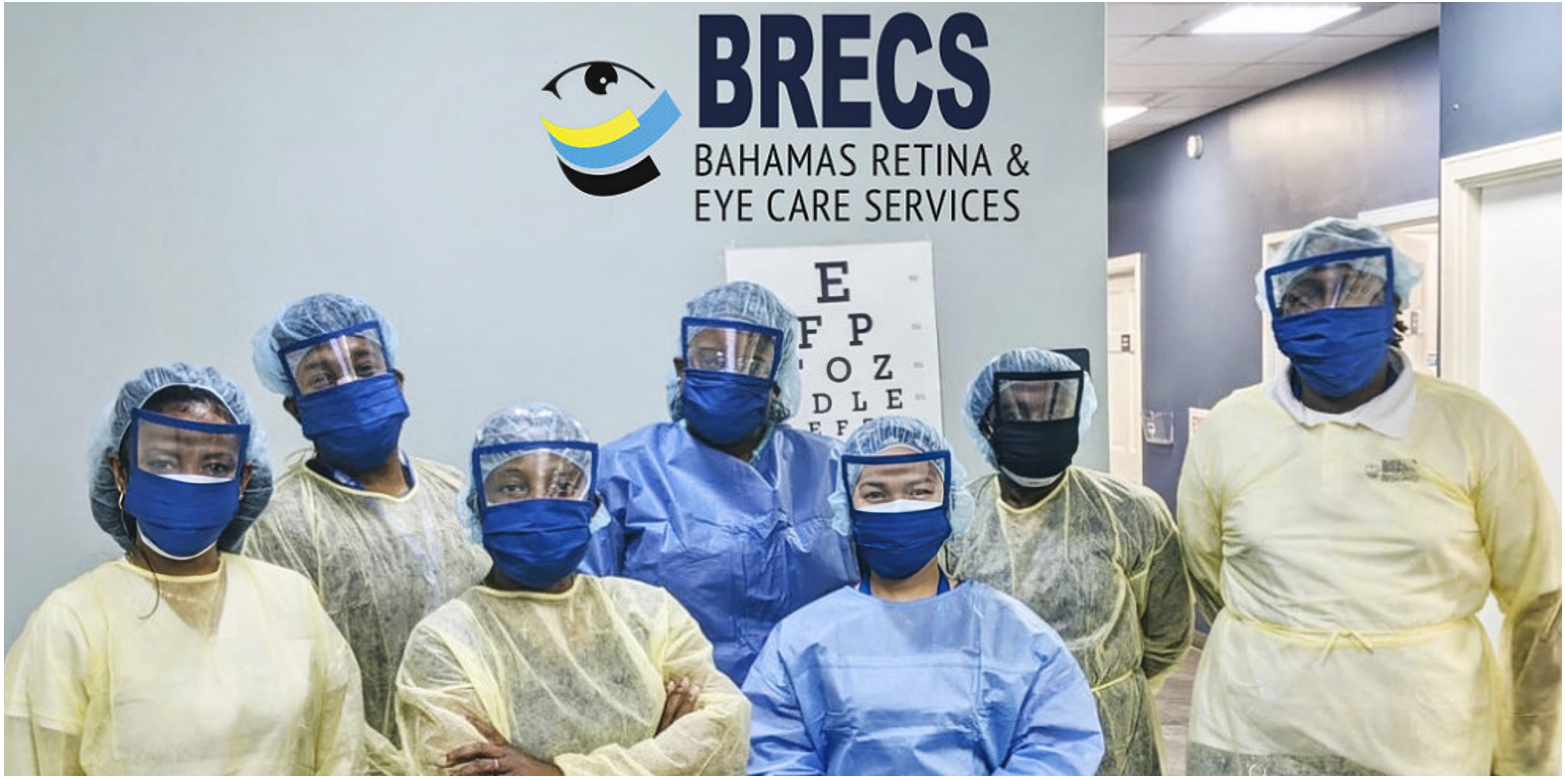
Eye care personnel at the Bahamas Retina and Eye Care Services during the COVID-19 pandemic. BAHAMAS

© DAWN HERMANNS CC BY-NC-SA 4.0

The rapid spread of COVID-19 and the subsequent strain on health care systems^1^ forced many hospitals and clinics to postpone or cancel non-urgent procedures to accommodate the influx of COVID-19 patients and prevent further virus transmission.^2^ Personnel were reassigned to COVID-related services in most countries^2^ and, as a result, many eye care services, including routine eye examinations and surgical procedures, were suspended or delayed.

The **International Agency for the Prevention of Blindness (IAPB) COVID-19 task force** was formed to support and offer guidance on eye care advocacy, programmes, and services during the pandemic. The goal of the global task force was to ensure that eye health services could continue in a manner that minimised the risk of COVID-19 transmission, while ensuring continuity of care for patients with eye conditions.

Building upon this work, the Fred Hollows Foundation and IAPB undertook a rapid review to assess the impact of COVID-19 on workforce, backlog, training, patients, and suspected new cases of sight loss. The focus was on cataract services, the leading cause of blindness globally. In total, 68 articles from 26 countries were identified and reviewed (Figure 1). Each article was reviewed, and the authors extracted the study type and key findings related to training, backlogs, recovery planning, and patient attendance, disaggregated by gender.

**Figure 1:**
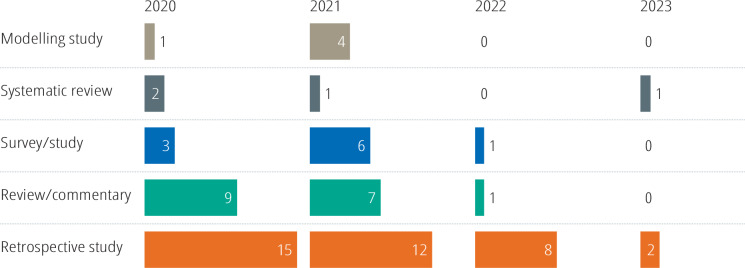
Studies included in rapid review, by study type.

## Key findings

### 1. The cataract backlog increased

Pre-pandemic, there were 94 million people worldwide living with sight loss due to unoperated cataract.^3^ During the COVID-19 pandemic, cataract surgery and other ophthalmological procedures experienced a significant decline. Ophthalmologists also grappled with heightened risks of COVID-19 infection and increased infection control policies for patients.^4^ These disruptions have led to backlogs in cataract operations, potentially posing long-term consequences for both patients and health care systems.^5^

### 2. Patient visits declined

Across all 26 countries, there was significant variation in the reduction of patient visits for all eye conditions, with some studies reporting no reduction and others reporting a complete halt (100% reduction). The average reduction across all studies was 61%. Patients presented with more advanced or severe disease and, in some areas, there was a reduction in urgent pathologies. This suggests patients delayed seeking care or surgery, which may have led to irreversible sight loss.

### 3. The situation got worse for women and girls

**Figure 2:**
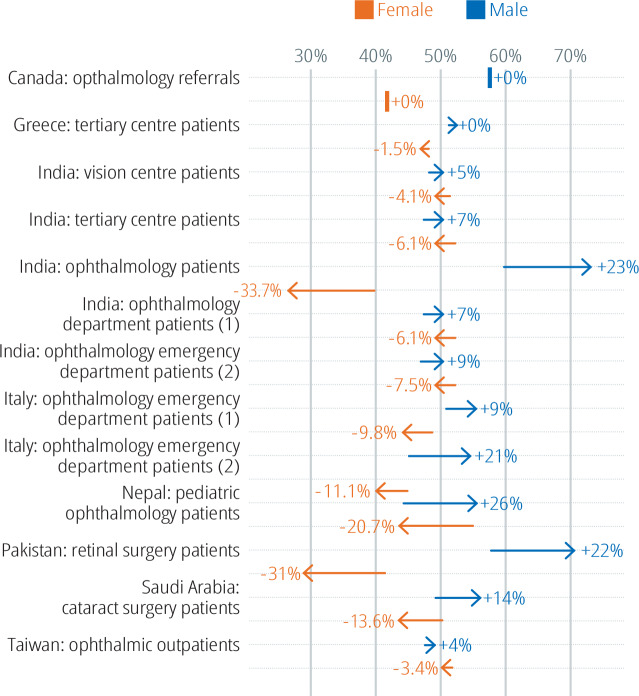
Percentage change in the proportion of male and female patients attending services once the COVID-19 shutdowns began, as reported by institutions.

Every study that looked at patient visits based on gender found that even fewer women and girls were accessing eye health services compared to men and boys. Prior to the pandemic, women and girls already had less access to eye health services in many countries due to various socioeconomic and cultural factors, but experienced more sight loss than men and boys, making up 55% of those affected globally.^6^ All studies that disaggregated patient attendance by sex reported a reduction in the proportion of women and girls that were accessing eye health services (Figure 2). This disparity was seen in every country providing data and was irrespective of the type of eye care being provided. This suggests that the pandemic made it harder for women and girls to access eye care, exacerbating existing inequities.

### 4. The situation got worse for children

Vision screening programmes in schools play a crucial role in identifying uncorrected refractive errors and other eye conditions in children across many regions. The global disruption caused by school closures led to the suspension of numerous school eye health programmes, depriving children of this vital opportunity for eye checks. Presently, there is limited information available regarding the resumption of these school-based eye health services; this is particularly urgent given the observed increase in myopia in various settings. A systematic review showed that access to paediatric eye care decreased during the pandemic, with an average drop of 67% in paediatric eye-related visits across studies,^7^ similar to the 61% reduction in patient visits (all ages) reported here. Delayed screening for retinopathy of preumaturity (ROP) was observed in West Bengal, India, along with increased odds of developing ROP during the COVID-19 pandemic.^8^

Disruptions to global nutrition programmes, and the potential for up to 117 million children in 37 low- and middle-income countries (LMICs) to have missed their routine vaccinations^9^ could result in an increase of vitamin A deficiency and measles – two causes of paediatric mortality and blindness.

### 5. People with other eye conditions may also experience long term consequences

**Diabetes complications.** The lengthy lockdowns due to the pandemic made it hard for people to get their usual diabetes care or to find out for the first time that they have diabetes. These lockdowns also made it more difficult for people to take care of their diabetes on their own and to seek treatment, most notably for sight-threatening diabetic retinopathy. Because of this, it is expected that more people may face problems related to diabetes, such as diabetic retinopathy, in years to come.^10^

**Myopia.** The COVID-19 pandemic has significantly impacted the progression of myopia, especially among children.^11^ The key factors that cumulatively contributed to an increase in myopia progression during the pandemic were:

1. Increased screen time and a shift towards remote learning and entertainment
2. Decreased outdoor activities
3. A change in learning environments from traditional classrooms to digital learning increased activities that focus on objects at close distances, such as using computers, tablets, and smartphones, and reduced distance viewing opportunities such as viewing a chalkboard or whiteboard, or observing a teacher at the front of the class.
4. Postponed eye care, potentially leading to delayed myopia diagnosis and treatment.

**Neglected Tropical Diseases (NTDs).** WHO interim guidance on NTDs advised a temporary halt on mass drug administration campaigns, active case detection, and epidemiological studies. This decision may have long term consequences in the goal to eliminate diseases such as onchocerciasis and trachoma.^12^

### 6. Training of eye care workers was affected

The COVID-19 pandemic significantly affected the training of eye care professionals. A notable reduction in elective surgery directly affected the hands-on learning experience essential for ophthalmology trainees, and higher surgical complication rates were observed in some areas compared to before the pandemic.^13^ However, the exploration of distance training methodologies, such as remote cataract surgical wet lab training, showcased the potential of leveraging technology to continue education.^1^

### 7. Services have not yet returned to COVID-19 levels

While some reports have suggested that the pandemic provided a chance to modernise and digitally transform service pathways, there is also an anticipated need to increase capacity to 150% of pre-pandemic workloads in some areas.^14^ While rapid resumption of services has been observed in some settings, it is unclear whether this has been sufficient to address the accumulated pandemic-related backlog.

## Conclusions and recommendations

The COVID-19 pandemic had a significant impact on health care services worldwide, with the de-prioritisation of non-emergency medical procedures and services resulting in substantial backlogs that have yet to be resolved. Our recommendations, based on this rapid review, are as follows:

1. **Carry out sector analysis.** Analyse the current eye health backlog to identify the specific challenges and capacity in each context. Identify where and how resources should be allocated to effectively address and reduce backlogs.
2. **Test approaches.** Establish a framework for developing and testing context-specific strategies in eye health care that strengthen systems, enhance the integration of eye care within both the health and non-health sectors, and increase the resilience of the eye health system against future shocks.
3. **Focus on inequities.** Initiate targeted programmes for women and girls and encourage gender-disaggregated data collection so that eye health inequities can be monitored. Resume and increase school eye health services to not only to continue regular eye care, but also to cater to the needs of those who missed out on essential eye screening and treatment during the pandemic.
4. **Increase funding.** Use evidence to advocate for increased funding from donors and governments to increase access to services.
